# EFAS/EAN survey on the influence of the COVID-19 pandemic on European clinical autonomic education and research

**DOI:** 10.1007/s10286-023-00985-3

**Published:** 2023-10-04

**Authors:** Alessandra Fanciulli, Magdalena Krbot Skorić, Fabian Leys, Diogo Reis Carneiro, Nicole Campese, Giovanna Calandra-Buonaura, Jennifer Camaradou, Giacomo Chiaro, Pietro Cortelli, Cristian Falup-Pecurariu, Roberta Granata, Pietro Guaraldi, Raimund Helbok, Max J. Hilz, Valeria Iodice, Jens Jordan, Evert C. A. Kaal, Anita Kamondi, Anne Pavy Le Traon, Isabel Rocha, Johann Sellner, Jean Michel Senard, Astrid Terkelsen, Gregor K. Wenning, Elena Moro, Thomas Berger, Roland D. Thijs, Walter Struhal, Mario Habek, the Collaborators of European Network of Neurological Autonomic Laboratories, Ivan Adamec, Arnaud Aerts, Leo L. R. Canta, Robert Shane Delamont, Frederik de Lange, Francesca Del Sorbo, Grazia Devigili, Rita Di Leo, Trang Dinh, Jacques-Olivier Fortrat, Janne Gierthmühlen, Martin Hemels, Julia Köhn, Thomas Krøigård, Axel Lipp, Andrea Maier, Lucio Marinelli, Anna Mazzeo, Ivan Milenkovic, Maciej Motyl, Maria Grazia Natali Sora, Judith Navarro-Otano, Kristian Bernhard Nilsen, Mario Oliveira, Petter Moe Omland, Giuseppe Pelliccioni, Yann Pereon, Roland Josef Resch, Camilla Rocchi, Frederic Roche, Joost Rutten, Beatriz Tijero-Merino, Marcin Tutaj, A. M. H. G. van der Heijden-Montfroy, Bas J. A. van Hoeve, Narender van Orshoven, Ruihao Wang, Werner J. Z’Graggen

**Affiliations:** 1 Department of Neurology, Medical University of Innsbruck, Anichstraße 35, 6020 Innsbruck, Austria; 2 Department of Neurology, University Hospital Centre, Zagreb, Croatia; 3 Faculty of Electrical Engineering and Computing, University of Zagreb, Zagreb, Croatia; 4 Department of Neurology, Faculty of Medicine, University of Coimbra, Coimbra, Portugal; 5 Neurology Department, Centro Hospitalar e Universitário de Coimbra, Coimbra, Portugal; 6 IRCCS Istituto delle Scienze Neurologiche di Bologna, Bologna, Italy; 7 Department of Biomedical and Neuromotor Sciences, University of Bologna, Bologna, Italy; 8 Patient Partner of the EAN Scientific Panel for Autonomic Nervous System Disorders, London, UK; 9 Faculty of Medicine and Health Sciences, University of East Anglia, Norwich, UK; 10 Autonomic Unit, National Hospital for Neurology and Neurosurgery, Queen Square, London, UK; 11 UCL Queen Square Institute of Neurology, Faculty of Brain Sciences, University College London, London, UK; 12 Department of Neurology, Faculty of Medicine, Transilvania University, Brasov, Romania; 13 Department of Neurology, Johannes Kepler University, Linz, Austria; 14 Icahn School of Medicine at Mount Sinai, New York, NY USA; 15 Department of Neurology, University Erlangen-Nuremberg, Erlangen, Germany; 16 German Aerospace Center, Cologne, Germany; 17 Medical Faculty, University of Cologne, Cologne, Germany; 18 Department of Neurology, Maasstad Ziekenhuis, Rotterdam, The Netherlands; 19 Department of Neurology, National Institute of Mental Health, Neurology and Neurosurgery, Budapest, Hungary; 20 Department of Neurology, Semmelweis University, Budapest, Hungary; 21 Department of Neurology, Centre Hospitalier Universitaire de Toulouse, Toulouse, France; 22 Cardiovascular Autonomic Function Lab, Faculty of Medicine and CCUL, University of Lisbon, Lisbon, Portugal; 23 Landesklinikum Mistelbach-Gänserndorf, Mistelbach, Austria; 24 Department of Neurology, Klinikum rechts der Isar, Technische Universität München, Munich, Germany; 25 Institut des Maladies Métaboliques et Cardiovasculaires, INSERM U 1297, Toulouse, France; 26 Department of Neurology, Aarhus University Hospital and Danish Pain Research Center, Aarhus University, Aarhus, Denmark; 27 Division of Neurology, Grenoble Institute of Neuroscience, Grenoble Alpes University, CHU of Grenoble, Grenoble, France; 28 Department of Neurology, Medical University of Vienna, Vienna, Austria; 29 Comprehensive Center for Clinical Neurosciences and Mental Health, Medical University of Vienna, Vienna, Austria; 30 Department of Neurology, Leiden University Medical Centre, Leiden, The Netherlands; 31 Stichting Epilepsie Instellingen Nederland (SEIN), Heemstede, The Netherlands; 32 Department of Neurology, University Hospital Tulln, Karl Landsteiner University of Health Sciences, Tulln, Austria; 33 Department of Neurology, University of Zagreb, School of Medicine, Zagreb, Croatia

**Keywords:** COVID-19 pandemic, Autonomic nervous system, Clinical autonomic education, Clinical autonomic research, e-Learning, Telemedicine

## Abstract

**Purpose:**

To understand the influence of the coronavirus disease 2019 (COVID-19) pandemic on clinical autonomic education and research in Europe.

**Methods:**

We invited 84 European autonomic centers to complete an online survey, recorded the pre-pandemic-to-pandemic percentage of junior participants in the annual congresses of the European Federation of Autonomic Societies (EFAS) and European Academy of Neurology (EAN) and the pre-pandemic-to-pandemic number of PubMed publications on neurological disorders.

**Results:**

Forty-six centers answered the survey (55%). Twenty-nine centers were involved in clinical autonomic education and experienced pandemic-related didactic interruptions for 9 (5; 9) months. Ninety percent (*n* = 26/29) of autonomic educational centers reported a negative impact of the COVID-19 pandemic on education quality, and 93% (*n* = 27/29) established e-learning models. Both the 2020 joint EAN–EFAS virtual congress and the 2021 (virtual) and 2022 (hybrid) EFAS and EAN congresses marked higher percentages of junior participants than in 2019. Forty-one respondents (89%) were autonomic researchers, and 29 of them reported pandemic-related trial interruptions for 5 (2; 9) months. Since the pandemic begin, almost half of the respondents had less time for scientific writing. Likewise, the number of PubMed publications on autonomic topics showed the smallest increase compared with other neurological fields in 2020–2021 and the highest drop in 2022. Autonomic research centers that amended their trial protocols for telemedicine (38%, *n* = 16/41) maintained higher clinical caseloads during the first pandemic year.

**Conclusions:**

The COVID-19 pandemic had a substantial negative impact on European clinical autonomic education and research. At the same time, it promoted digitalization, favoring more equitable access to autonomic education and improved trial design.

**Supplementary Information:**

The online version contains supplementary material available at 10.1007/s10286-023-00985-3.

## Introduction

With the rise of herd immunity, four years into the coronavirus disease 2019 (COVID-19) pandemic, diminishing numbers of newly diagnosed COVID-19 cases and deaths are registered worldwide [36]. Notwithstanding, the COVID-19 pandemic brought major changes to our society and healthcare systems, the consequences of which are still being dealt with. Beyond the global drug [19] and medical device shortages [29], supply chain disruptions [6, 35], and increased healthcare workers’ burden [18, 22, 26], the COVID-19 pandemic directly affected clinical neurological practice in multiple ways. In the field of autonomic nervous system disorders, many referral centers were forced throughout Europe to stop their activities due to pandemic containment measures, with major consequences on the quality and continuity of autonomic healthcare provision [4, 12]. On the other hand, both COVID-19 and, to a lesser extent, COVID-19 vaccines were reportedly associated with new diagnoses or significant worsening of previously diagnosed cardiovascular autonomic disorders, increasing the number of individuals requiring specialized autonomic care [12, 13, 30].

Studies among neurology trainees and teachers highlighted how the COVID-19 pandemic negatively influenced not only clinical practice, but also bedside learning and training of neurological skills [9, 15, 32]. Likewise, the pandemic outbreak severely impacted neurological research, with several non-COVID-19-related clinical trials being put on hold, not initiated at all, or undergoing substantial amendments to mitigate the pandemic-related recruitment losses and drop-outs [2, 5, 9].

It is however still unknown how and to what extent the COVID-19 pandemic influenced the educational and research activities in neurology subspecialties with limited availability of referral centers across European countries, such as the autonomic field. To answer this question, the European Federation of Autonomic Societies (EFAS) and the Scientific Panel for Autonomic Nervous System Disorders of the European Academy of Neurology (EAN) launched a joint web-based survey among the European neurological autonomic centers investigating the influence of the COVID-19 pandemic on clinical autonomic education and research.

## Methods

### Survey participants

A comprehensive description of the survey methodology has been previously published [16]. Briefly, we invited all the directors of neurology-driven and interdisciplinary (i.e., with at least one neurologist in the core team) European autonomic centers to complete a web-based survey between September and November 2021. Up to three reminders were sent to nonrespondents prior to the survey closure.

### Questionnaire

The questionnaire covered the following topics (see full text in the Supplementary Material):

- Demographic information of the survey respondents and characteristics of the autonomic center, including equipment, staff and pre-pandemic-to-pandemic caseload;
- Influence of the COVID-19 pandemic on autonomic educational activities and lessons learned for an improved autonomic educational offer;
- Influence of the COVID-19 pandemic on clinical autonomic research, time availability for scientific writing, and lessons learned for an optimized autonomic research practice.

### COVID-19 pre-pandemic-to-pandemic proportion of junior participants in international neurological congresses

To understand the effect of in-person versus virtual or hybrid congress formats on the proportion of junior participants attending the annual general neurology (i.e., EAN) and autonomic subspecialty congresses (i.e., EFAS), we asked the EAN head office and the organizers of the 2019–2022 EFAS congresses about the overall number of congress participants and registered junior participants (residents, research fellows, and undergraduate students).

### COVID-19 pre-pandemic-to-pandemic publication output in different neurological subspecialties

For comparison purposes, we searched the PubMed database for the 2017–2022 number of publications/year on autonomic versus other main neurological disorders and subspecialties using the search terms reported in the Supplementary Material. The count included all types of manuscripts without language restrictions. The annual percentage change in the number of publications was plotted separately for each neurology subspecialty for visual comparison.

### Statistical analysis

Qualitative variables were summarized in frequencies (percentages) and compared using the chi-square or Fisher exact test. Quantitative variables were summarized in median values (25th; 75th percentile) or mean ± standard deviation. The Mann–Whitney *U*-test was used for comparing non-normally distributed quantitative variables, the *t* test for normally distributed ones. Associations between variables were tested with the Spearman’s correlation coefficient. All statistical analyses were performed with IBM-SPSS (version 25). Two-sided *P* values < 0.05 were considered statistically significant.

Firstly, we performed a descriptive analysis of the characteristics of the survey respondents and autonomic centers involved in autonomic education compared with those respondents who were not. We then assessed the influence of the COVID-19 pandemic on the availability and quality of clinical autonomic education. Whenever the number of respondents was more than eight per subgroup, we determined whether the respondent or center characteristics, including their geographical localization [16], were significantly associated with any educational outcome measure.

Secondly, we summarized the characteristics of the centers actively involved in clinical autonomic research versus those that were not. Afterwards, we analyzed how the COVID-19 pandemic influenced the research performance of the respondents, including time availability for paper and grant writing. Whenever an adequate sample size was available (i.e., more than eight respondents per subgroup), we determined whether any specific respondent or center characteristic was significantly associated with the reported changes in research practice.

Open-ended questions on lessons learned from the COVID-19 pandemic for an improved autonomic education and research practice were analyzed with a semiquantitative technique.

Thirdly, we documented the pre-pandemic-to-pandemic percentage of junior participants in the annual EAN and EFAS congresses, and the annual percentage change in the number of PubMed publications on autonomic versus other main neurological disorders.

### Data availability statement

The first and the last authors take full responsibility for the integrity of data and agree to share any de-identified data not published herewith upon reasonable request from any qualified investigator.

## Results

### Survey respondents

Forty-six out of 84 (55%) directors of clinical autonomic centers from 22 European countries answered the survey. Detailed information on the respondents and center characteristics has been previously published [16]. Briefly, the respondents and autonomic centers showed homogeneous characteristics across Europe, but their geographical distribution was skewed towards Northern/Western European countries compared with Southern/Eastern European ones [16].

Twenty-nine (63%) survey respondents were reportedly involved in autonomic educational activities, without differences in terms of demographic characteristics, years of clinical practice, or geographical localization with respect to respondents not involved in autonomic education (Table 1). The percentage of autonomic centers closed during the COVID-19 pandemic and the length of such closures also did not differ between centers that were or were not involved in autonomic education (Table 1). Notwithstanding, centers involved in autonomic education performed a higher number of tilt-table tests (TTT) (*p* = 0.016) and autonomic outpatient visits (*p* = 0.034) in the first pandemic year compared with those not involved in educational activities (Table 1).

**Table 1:** Characteristics and COVID-19 pre-pandemic-to-pandemic clinical caseloads of the European autonomic centers involved versus non-involved in clinical autonomic education and research

Variable	Clinical autonomic education			Clinical autonomic research	
	Involved labs	Non-involved labs	*p* value	Involved labs	Non-involved labs
	(*n* = 29)	(*n* = 17)		(*n* = 41)	(*n* = 5)
Head of the laboratory					
Female (%)	9 (31%)	9 (52.9%)	0.212	17 (41.5%)	1 (20%)
Age (years)					
30–39	5 (17.2%)	3 (17.6%)	0.689	8 (19.5%)	0
40–49	15 (51.7%)	6 (35.3%)		20 (48.8%)	1 (20%)
50–59	7 (24.1%)	7 (41.2%)		10 (24.4%)	4 (80%)
60–69	2 (6.9%)	1 (5.9%)		3 (7.3%)	0
Years into practice					
Resident	1 (3.4%)	1 (5.9%)	0.235	2 (4.9%)	0
Junior consultant (0–4 years)	0	1 (5.9%)		1 (2.4%)	0
Consultant (5–9 years)	3 (10.3%)	1 (5.9%)		4 (9.8%)	0
Senior consultant (10–19 years)	13 (44.8%)	3 (17.6%)		15 (36.6%)	1 (20%)
> 20 years	12 (41.4%)	11 (64.7%)		19 (46.3%)	4 (80%)
Geographical localization*	20 (69%):9 (31%)	10 (59%):7 (41%)	0.534	25 (61%):16 (39%)	5 (100%):0 (0%)
Cumulative number of staff members	9 (7–12)	7 (6–13)	0.755	8 (6–12)	10 (8–24)
No. of TTT/year before COVID-19	150 (55; 300)	50 (42; 175)	0.06	120 (50; 252)	40 (24; 200)
No. of outpatient visits/year before COVID-19	220 (150; 400)	113 (65; 250)	0.06	200 (100; 340)	100 (;)
No. of inpatient visits/year before COVID-19	20 (5; 95)	50 (1; 125)	0.79	25 (5; 118)	4 (;)
No. of TTT in the 1st pandemic year	**66 (30; 161)**	**20 (1; 75)**	**0.016**	60 (20; 118)	15 (5; 145)
No. of outpatient visits in the 1st pandemic year	**140 (50; 235)**	**45 (15; 90)**	**0.034**	110 (40; 210)	80 (;)
No. of inpatient visits in the 1st pandemic year	10 (2; 43)	7 (0; 38)	0.438	10 (1; 46)	3 (;)
Percentage reduction in TTT/year	−50% (−67%; −27%)	−47% (−92%; −20%)	0.45	−50% (−75%; −24%)	−29% (−80%; −10%)
Percentage reduction in outpatient visits/year	−43% (−61%; −10%)	−50% (−72%; −10%)	0.563	−50% (−66%; −18%)	−20% (;)
Percentage reduction in inpatient visits/year	−50% (−86%; −14%)	−56% (−98%; −23%)	0.41	−53% (−88%; −20%)	−35% (;)
No. of inhabitants in the referral area					
≤ 500,000	6 (20.7%)	6 (35.3%)	0.651	9 (22%)	3 (60%)
500,000–1,000,000	13 (44.8%)	7 (41.2%)		18 (43.9%)	2 (40%)
1,000,000–5,000,000	9 (31%)	3 (17.6%)		12 (29.3%)	0
> 5,000,000	1 (3.4%)	1 (5.9%)		2 (4.9%)	0
Closure of TTT labs during the pandemic	21 (75%)	10 (58.8%)	0.326	29 (72.5%)	2 (40%)
Length of closure of the TTT labs, in months	2 (2; 5)	5 (4; 9)	0.072	5 (2; 9)	2 (;)
Closure of outpatient clinics during the pandemic	17 (60.7%)	10 (58.8%)	1	25 (62.5%)	2(40%)
Length of closure of outpatient clinics, in months	2 (2; 5)	5 (2; 9)	0.486	5 (2; 9)	2 (;)
Closure of inpatient clinics during the pandemic	12 (42.9%)	8 (47.1%)	1	20 (50%)	0
Length of closure of inpatient clinics, in months	2 (2; 8)	5 (2; 9)	0.348	2 (2; 9)	–

Comparisons between centers involved and non-involved in clinical autonomic research were not performed due to the low number of centers not involved in research (*n* = 5)

Statistically significant differences are highlighted in bold

No./n , number; TTT, tilt-table tests

^*^Northern/Western Europe versus Southern/Eastern Europe

Most survey respondents were involved in autonomic research (89%, *n* = 41), mainly focused on autonomic failure in movement disorders, postural orthostatic tachycardia syndrome and reflex syncope (Table 1) [16]. No subgroup comparison was therefore performed.

### Impact of the COVID-19 pandemic on clinical autonomic education in Europe

**Fig. 1:**
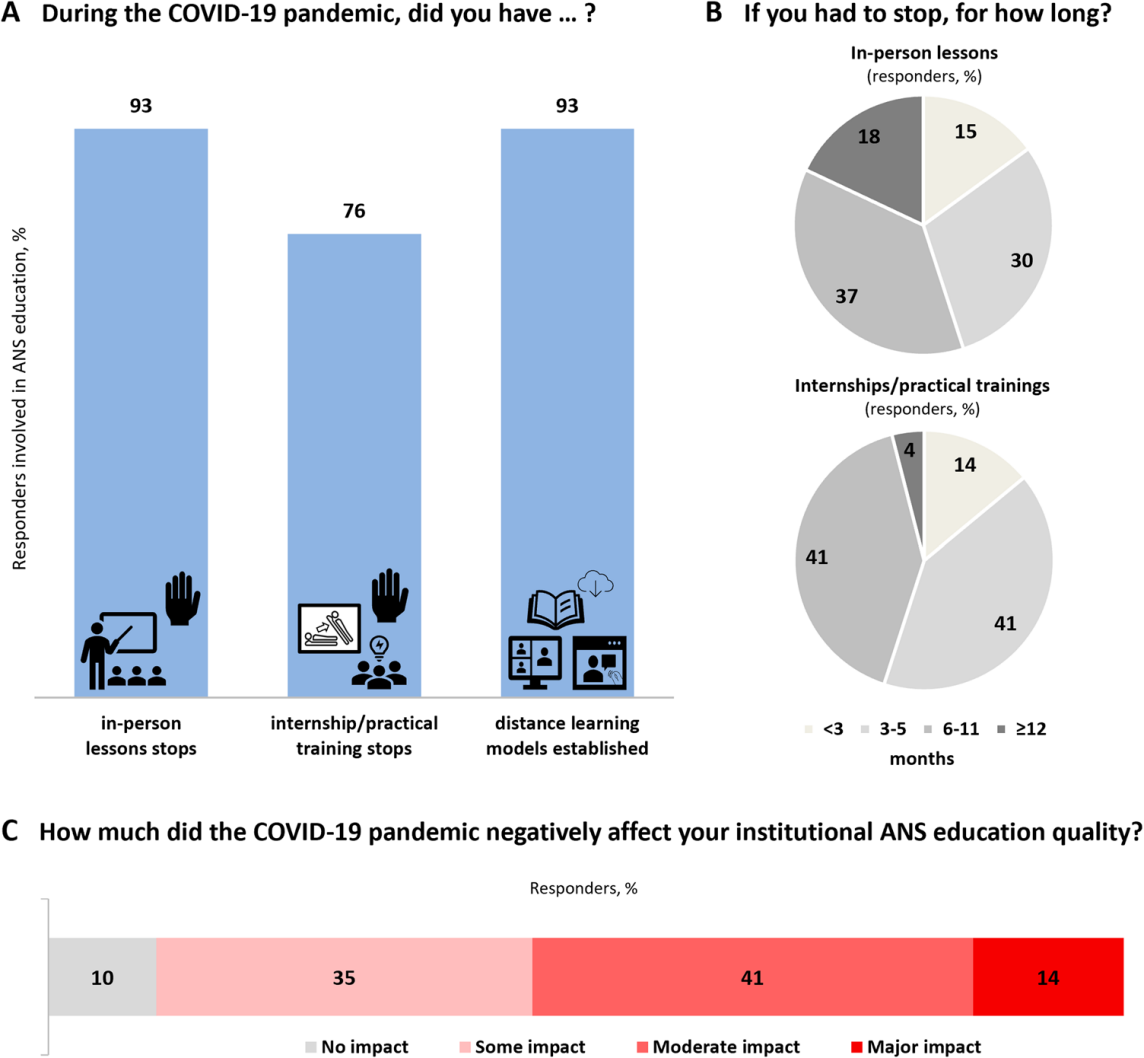
Type (**A**) and length (**B**) of changes in clinical autonomic educational activities from the beginning of the COVID-19 pandemic in the institutions of the European clinical autonomic centers. **C** The estimated impact of the COVID-19 pandemic on the overall quality of clinical autonomic education in the opinion of the survey respondents. Centers with in-person lesson stops for more than 6 months had a significantly lower number of TTT (74 ± 81 versus 231 ± 223, *p* = 0.037) and autonomic inpatient admissions during the first pandemic year (15 ± 28 versus 45 ± 38, *p* = 0.036), as well as longer autonomic function laboratory [5 (4; 9) versus 2 (2; 3) months, *p* = 0.013] and outpatient clinic [5 (5; 9) versus 2 (2; 2) months, *p* = 0.004] closures. Centers reporting internship training stops for longer than 6 months had a significantly higher cumulative number of staff members (11 ± 4 versus 7 ± 4, *p* = 0.038) and bigger reduction in the number of TTT during the first pandemic year (−57 ± 29% versus −30 ± 25%, *p* = 0.027). Participants who reported a moderate to severe negative effect of the COVID-19 pandemic on clinical autonomic education were more frequently from Southern/Eastern European than Northern/Western European countries (89% versus 40%, *p* = 0.020), had greater reductions in the number of autonomic outpatient visits during the first pandemic year (−49 ± 25% versus −23 ± 28%) and longer autonomic function laboratory closures [5 (2; 9) versus 2 (2; 3) months, *p* = 0.049]

**Fig. 2:**
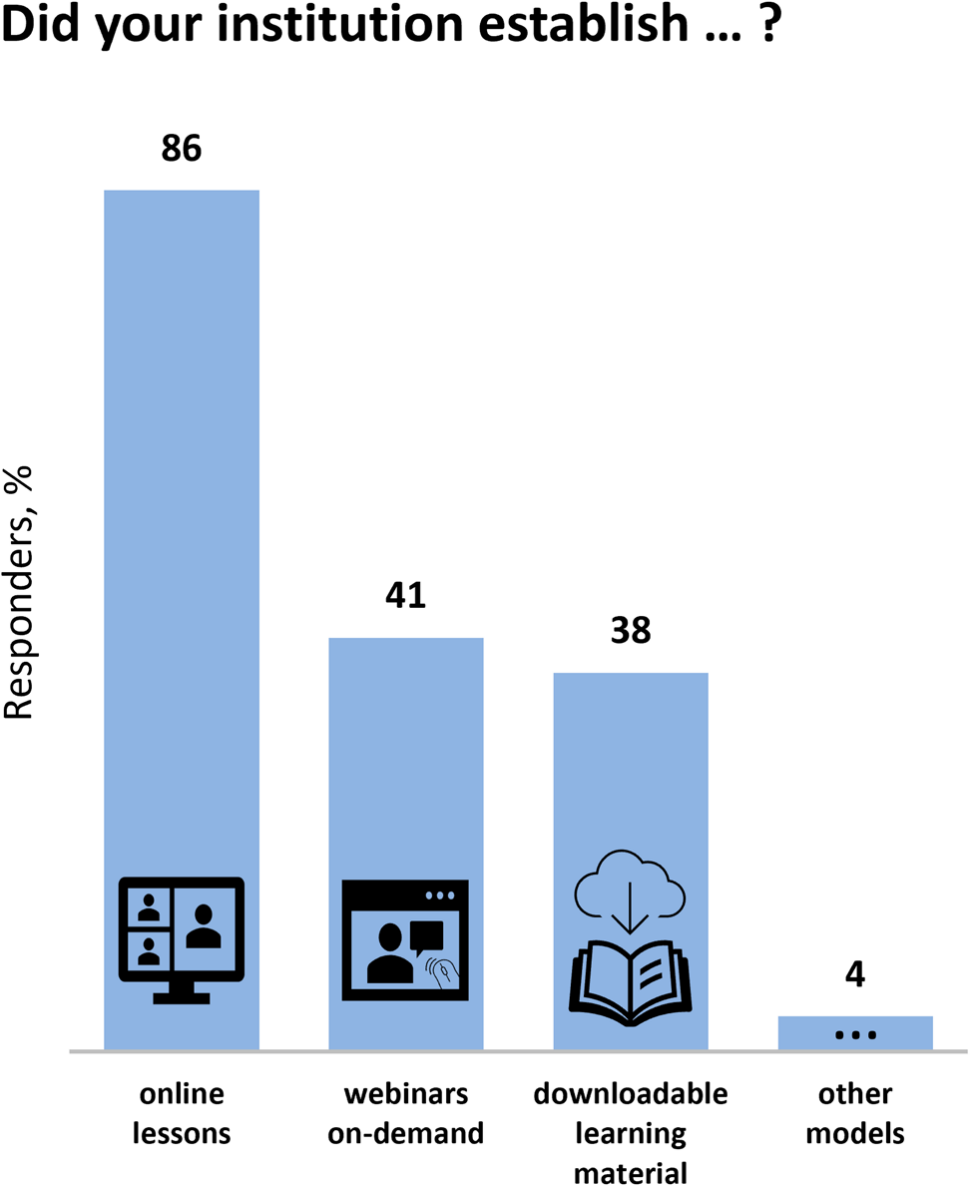
Types of distance learning models established in the institutions of the European clinical autonomic centers involved in autonomic education since the COVID-19 pandemic begin. Other models of distance learning included interactive case presentations

Since the pandemic outbreak, 93% (*n* = 27/29) of autonomic centers involved in education were forced to stop in-person teaching for a median of 9 (5; 9) months (Fig. 1). Seventy-six percent (*n* = 22/29) of centers involved in autonomic education also interrupted internship and practical training programs for 5 (5; 9) months (Fig. 1). In response to the COVID-19 pandemic-related educational barriers, most centers (93%, *n* = 27/29) switched to e-learning formats (Fig. 2). Notwithstanding, 90% of survey respondents involved in clinical autonomic education agreed that the COVID-19 pandemic had a negative effect on the overall quality of their institutional educational offer; 55% (*n* = 16/29) judged such effect as moderate to severe (Fig. 1C).

### Lessons learned from the COVID-19 pandemic for an improved clinical autonomic education

Six respondents made suggestions regarding possible strategies to raise the quality of clinical autonomic education in the future. They recommended the integration of online courses and on-demand webinars in medical school curricula and the enrichment of neurology residency programs with rotations in autonomic units, autonomic case-series classes, and in-person or remote autonomic video clinic attendance.

### COVID-19 pre-pandemic-to-pandemic proportion of junior participants in the annual EAN and EFAS congresses

**Fig. 3:**
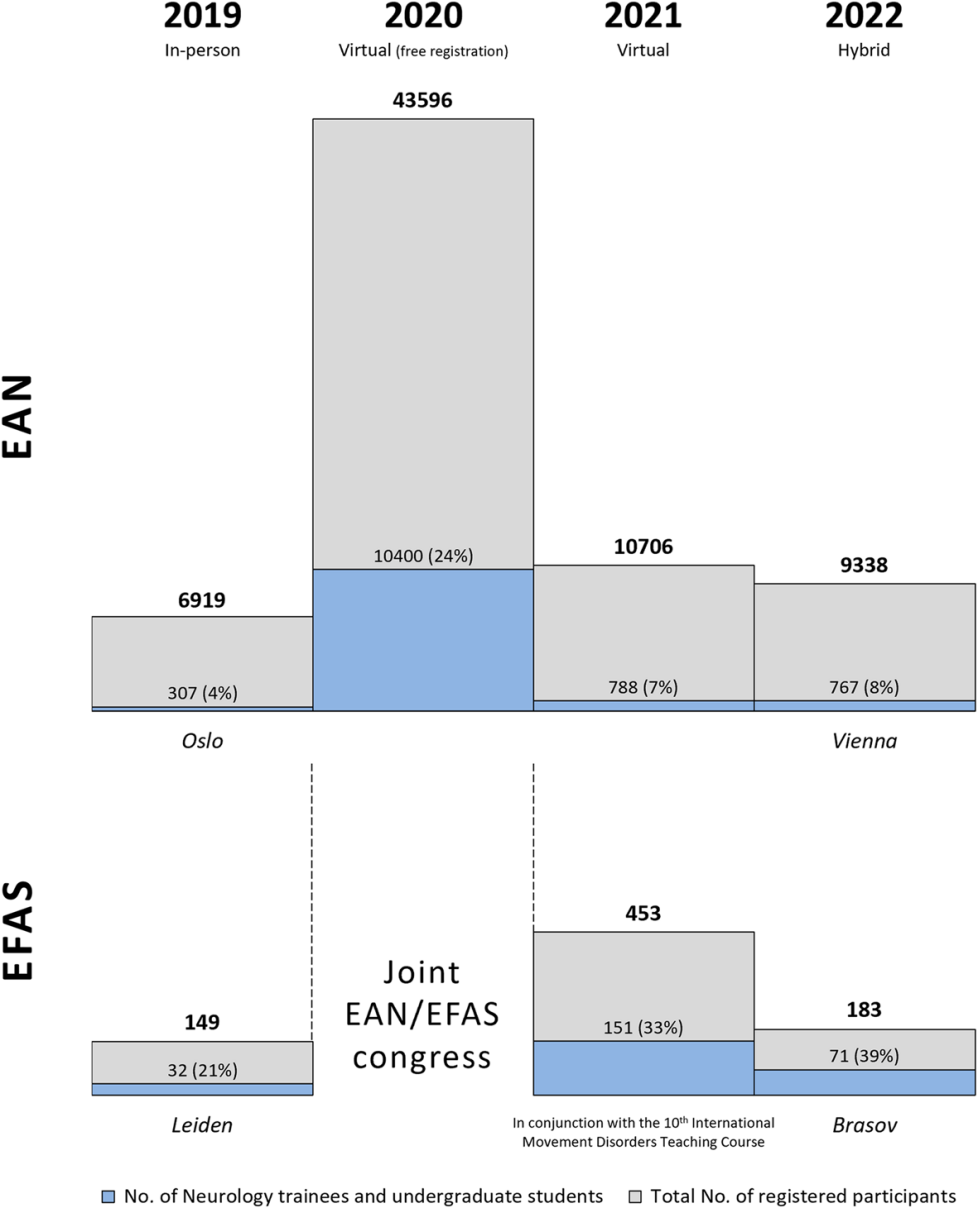
COVID-19 pre-pandemic-to-pandemic number of neurology trainees and undergraduate students participating in the annual congresses of the European Academy of Neurology (EAN) and European Federation of Autonomic Societies (EFAS). The EAN congress program entailed two sessions on autonomic nervous system topics in 2019, five in 2020, three in 2021, and two in 2022

Following the world-wide lockdown in March 2020, the EAN rapidly converted the planned annual congress into a fully virtual event with free registration for all attendees. This was an EAN–EFAS joint congress and marked a substantial increase in the number of registered junior participants (Fig. 3) from European, North American, South American, and Asian countries [33]. In 2021, the EAN and EFAS annual congresses were organized in a virtual format, with reduced registration fees for EAN neurology trainees and waved fees for EFAS trainees and EAN/EFAS undergraduate students. Both congresses ultimately achieved higher percentages of registered junior participants than in pre-pandemic years (Fig. 3). Such rising trend was confirmed in 2022, when both the EAN and EFAS opted for a hybrid congress format (Fig. 3).

### Impact of the COVID-19 pandemic on clinical autonomic research in Europe

**Fig. 4:**
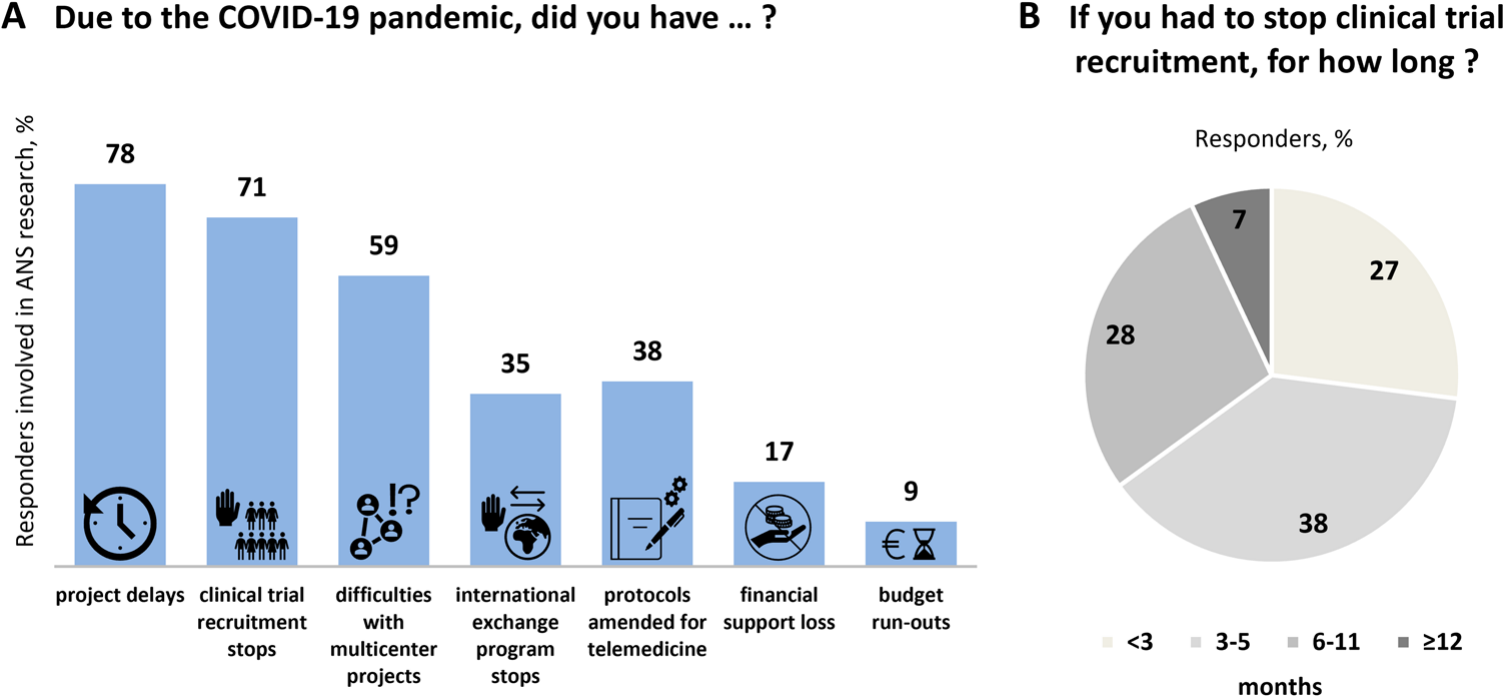
Impact of the COVID-19 pandemic on clinical autonomic research in Europe (**A**), including cumulative trial recruitment stop duration (**B**). Centers reporting trial recruitment stops for more than 6 months had a significantly lower number of autonomic outpatient visits during the first pandemic year [48 (25; 123) versus 125 (85; 274), *p* = 0.045] compared with those whose trial recruitment stops lasted 6 months or less. Survey respondents, who amended their study protocols for telemedicine had a significantly higher number of TTT both in pre-pandemic years [250 (50; 400) versus 100 (47; 195), *p* = 0.015] and during the first pandemic year (194 ± 219 versus 63 ± 72, *p* = 0.039), as well as higher numbers of autonomic inpatient admissions during the first pandemic year [28 (9; 96) versus 5 (0; 24), *p* = 0.022] compared with those who did not implement telemedicine in their research protocols

Since the pandemic onset, 78% of European autonomic centers involved in research activities experienced project delays, with clinical trial recruitment stops for 5 (2; 9) months (Fig. 4A, B), difficulties in managing multicenter projects (59% of respondents, *n* = 23/39), and international scientific exchange programs (35%, *n* = 14/40). As a result, 17% (*n* = 7/41) of researchers faced financial losses and 9% (*n* = 3/33) budgetary runouts. Thirty-eight percent (*n* = 15/40) of autonomic researchers were able to amend their study protocols, embedding telemedicine-based visits and study outcome measures (Fig. 4).

**Fig. 5:**
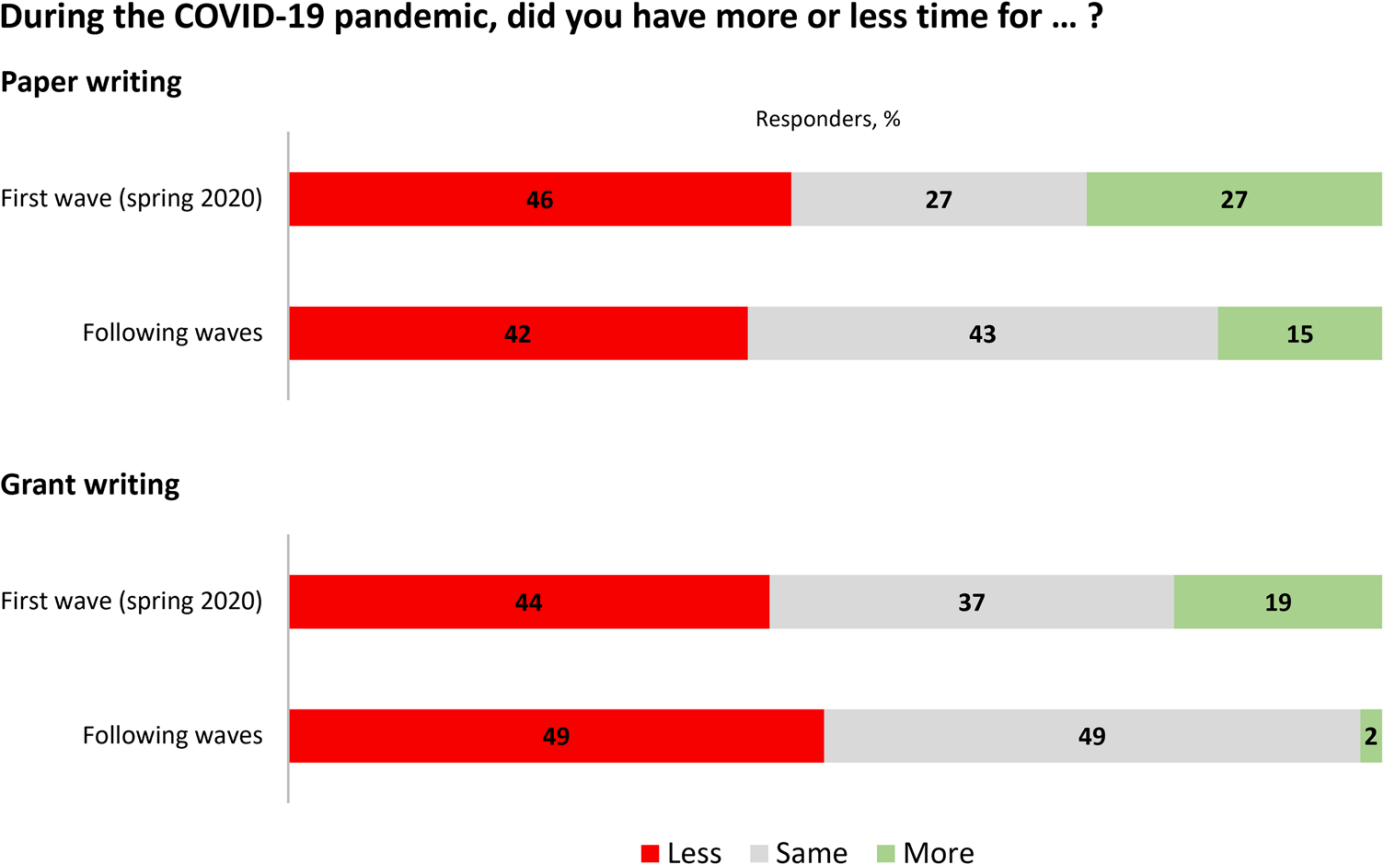
Impact of the COVID-19 pandemic on time availability for paper and grant writing in the experience of the survey respondents involved in clinical autonomic research. We found no association between any respondent or autonomic center characteristic and the COVID-19 pre-pandemic-to-pandemic change in time availability for paper writing. Likewise, we observed no difference in the respondents or center characteristics between respondents reporting increased or diminished time available for grant writing during the first pandemic wave, but respondents reporting less time for grant writing during the following waves belonged to centers with longer autonomic function laboratory closures (6 ± 4 versus 4 ± 3 months, *p* = 0.036)

When specifically asked about the availability of time to write scientific papers during the first COVID-19 pandemic wave, 46% (*n* = 19/41) of autonomic researchers had less time, while 27% (*n* = 11/41) had more time than before the pandemic (Fig. 5). During the following waves, the percentage of respondents who had less time than usual for paper writing remained similar (42%, *n* = 17/41), but the percentage of those who reported to have more time for publishing than usual dropped to 15% (*n* = 6/41, Fig. 5). Such time constraints were reportedly even more impactful on the preparation of research grant proposals (Fig. 5).

### Lessons learnt from the COVID-19 pandemic for improved clinical autonomic research practice

Eleven respondents shared their opinion on novelty elements and needs raised by the COVID-19 pandemic in autonomic research settings; eight considered the introduction of online meetings a cost- and time-effective strategy for lowering the networking barriers among autonomic centers. Two respondents stressed the importance of securing adequate amounts of research time, financial, and infrastructural support for clinical autonomic research. Three respondents finally highlighted how the COVID-19 pandemic facilitated telemedicine implementation in research protocols, overall adding flexibility to clinical trial design.

### Pre-pandemic-to-pandemic number of PubMed publications on autonomic versus other neurological disorders

**Fig. 6:**
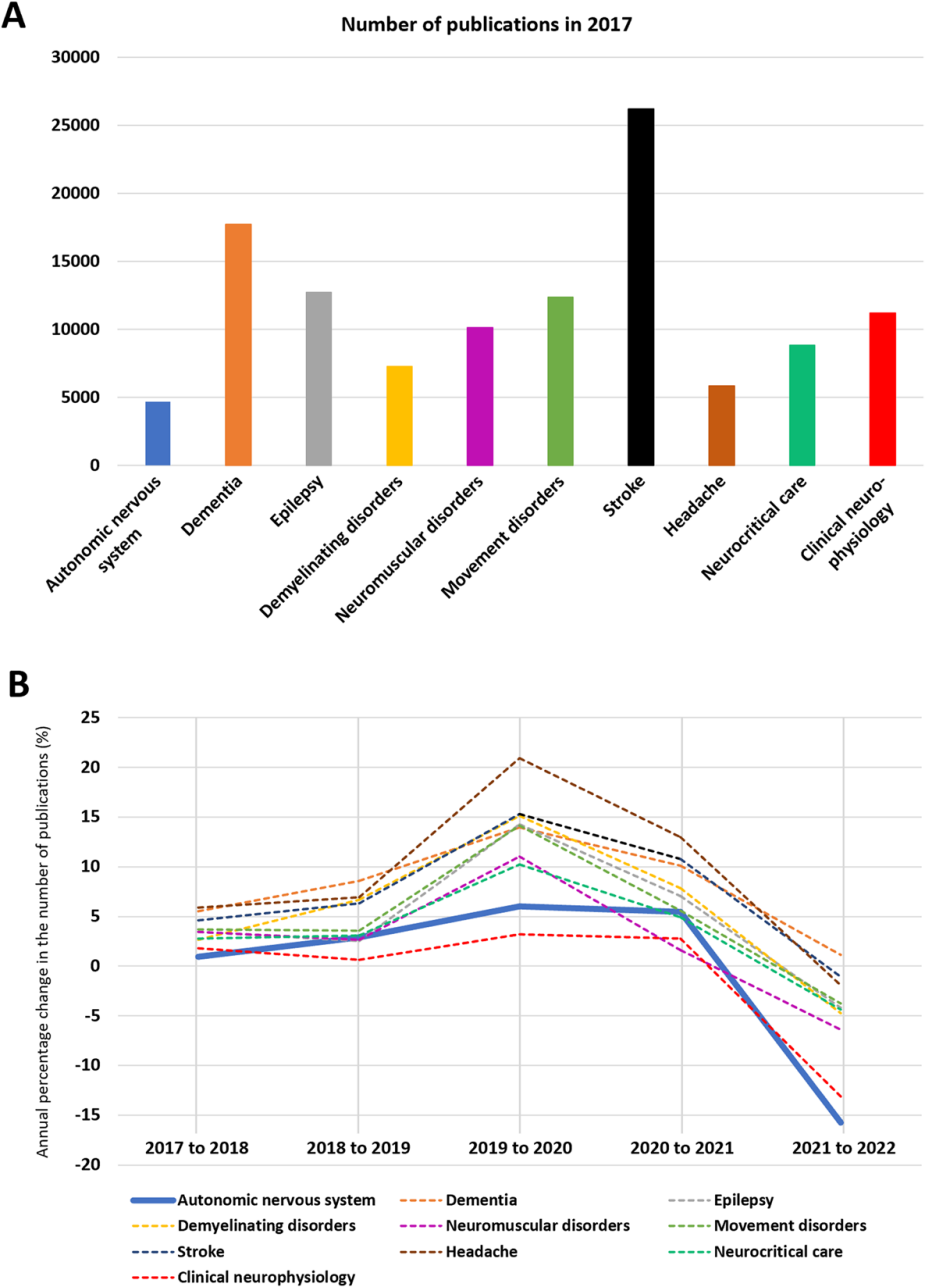
Cumulative number of publications in a pre-COVID-19 pandemic year (2017) on autonomic nervous system versus other frequent neurological disorders and neurology subspecialties (**A**). **B** The COVID-19 pre-pandemic-to-pandemic annual percentage change in the number of publications on autonomic topics (blue line) compared with other main neurological disorders and subspecialties (dotted lines)

Before the pandemic, the number of PubMed publications on autonomic disorders was the lowest among several other major neurological subspecialties (*n* = 4675, Fig. 6A) but showed comparable annual percentage increases [+1% versus +3% (3%; 5%), Fig. 6B].

In 2020, the annual number of publications increased substantially in all neurology subspecialties [+14% (11%; 15%)]. In the autonomic field, however, the annual percentage increase was substantially lower (+6%) compared with other fields, such as headache (+21%), multiple sclerosis (+15%), or stroke (+15%, Fig. 6B). In 2021, the annual percentage increase in the number of publications attenuated in all neurology subspecialties [+7% (4%; 10%)], including autonomic nervous system disorders (+5%). Except for the dementia field, in 2022, the number of publications diminished by 4% (−2%; −6%) in all neurology fields compared with 2021. This reduction was most prominent for autonomic (−16%) and clinical neurophysiology (−13%) research topics.

## Discussion

This survey highlighted a previously underestimated, substantial impact of the COVID-19 pandemic on clinical autonomic education in Europe.

We found that centers involved in clinical autonomic education maintained higher clinical caseloads than non-involved centers during the first pandemic year. Educational centers were possibly national referral centers enabled to continue working and/or receive referrals from other peripheral centers, which were closed during the lockdown. Among the centers involved in clinical autonomic education, those with longer teaching interruptions were also those with major pandemic-related reductions in the autonomic outpatient and inpatient metrics. This may indicate increased difficulties in controlling the pandemic spread at a local level, or alternatively reflect stricter lockdown policies in some European countries [7, 17, 37].

Respondents from Southern/Eastern European countries pinpointed a more severe pandemic-related autonomic educational gap than those from Northern/Western European countries. Such a gap likely affected trainees of two to three subsequent academic years, who may have been also exposed to increased risks of anxiety, depression, and burnout [11] and now need to close their knowledge gaps with individual efforts. To this end, the survey respondents indicated e-learning educational formats as a useful tool to break down the geographical barriers to clinical autonomic education. Digital educational formats may, in fact, promote more equitable access to education in neurology and its subspecialties, as highlighted by the increasing number of trainees, especially those from lower-income countries participating in the EAN and EFAS annual congresses since the introduction of less expensive virtual formats. At the same time, in-person participation of younger colleagues in international congresses should be actively encouraged with dedicated travel grants, reduced registrations fees, and invitations to give oral presentations to promote their engagement in professional networks [21, 34].

The survey respondents also highlighted multiple interferences of the COVID-19 pandemic with European autonomic research. Early during the pandemic, lawmakers may have deemed the risk–benefit ratio of research activities unfavorable in the short term. The long-term effects of missed or delayed research progress on healthcare quality are, however, yet to be quantified, especially considering that autonomic disturbances may frequently develop or worsen within the so-called post-COVID-19 condition [12, 25, 30]. To this end, it is concerning that most survey respondents reported diminished time available for scientific writing since the pandemic onset, which further decreased during the following pandemic waves. The fact that many regular duties had to be carried on with additional COVID-19 safety constraints possibly raised the overall individual workload to a critical level. This is mirrored by the number of PubMed publications on autonomic nervous system disorders, which showed the least annual percentage increase with respect to other neurological subspecialties during the first two pandemic years and the most pronounced reduction in 2022. Similar negative trends were observed in other subspecialties relying on elective diagnostic workups, such as clinical neurophysiology.

In analogy to the autonomic educational outcomes, we found that centers interrupting their trial recruitment for longer times also had lower clinical performances during the first pandemic year. These centers might have been hit “harder” in terms of local pandemic spread, or staff members might have suffered severe COVID-19 forms or developed post-COVID-19 disturbances, diminishing their working capability for some time. On the other hand, some institutions might have been better able to implement pandemic-mitigating strategies, as shown by the fact that centers implementing telemedicine in their research activities also performed more autonomic assessments and visits during the first pandemic year.

The pandemic-related healthcare digitalization, thus, promoted resilience not only in clinical autonomic practice [12, 31], but also in research, reshaping clinical trial design in several ways. Several observational studies—and even parts of interventional studies—are now often run in a decentralized way, with comparable recruitment rates and degrees of patient satisfaction [23]. For this purpose, video-based versions of conventional motor rating scales have been developed [14], eventually highlighting a high intra-individual variability in motor performance in home settings, which would have otherwise remained undetected with traditional hospital-based assessments [10, 14]. Telemonitoring tools are being increasingly integrated in clinical studies [1], further shifting the focus towards rater-independent outcome measures [27, 28]. Many organizational study meetings are now also run in a virtual format, reducing organizational costs. This is particularly important in the setting of rare autonomic disorders, where lower operational costs may encourage industry investments.

Research digitalization may also provide other types of benefits to the scientific community. Faster information exchange through online meetings fosters multicenter cooperation. Lowered need for business traveling has the potential of promoting individual productivity, work–life balance, and gender parity, facilitating child and other familial care duties. The decreased number of both business flights and patients’ travels to study centers may ultimately reduce the ecological footprint of research activities.

The present study has some limitations. First, the results are based on the information provided by the survey respondents, while consulting source data was beyond the scope of the present work. The survey respondents were, however, directors of autonomic centers with experienced insights into the European clinical autonomic landscape. The additional objective outcome measures considered in the present study, such as the pre-pandemic-to pandemic number of junior participants attending international congresses and the number of scientific publications on autonomic versus other neurology subspecialty topics, were also aligned with the respondents’ views. Second, our study might have underestimated the impact of the pandemic on clinical autonomic education and research in regions without available autonomic centers or in those identified centers that did not complete the survey despite the reminders sent. In order to reduce inequity in clinical autonomic education and research across Europe, both EFAS and the EAN should work on further promoting the inclusion of currently underserved countries and colleagues in their professional network and future initiatives. Third, the keywords used for the PubMed search on the pre-pandemic-to-pandemic number of publications across neurology subspecialties might have missed publications including autonomic outcome measures among the secondary objectives or studies investigating autonomic disturbances in common neurological conditions, such as movement disorders, stroke, or epilepsy, may have been counted multiple times in the PubMed search. To overcome this potential methodological bias, we based our comparative analysis on the annual percentage change in the number of publications per neurology subspecialty. Fourth, we were unable to quantify the monetary impact of the COVID-19 pandemic on academic research funding. Studies in other medical specialties reported pandemic-related financial losses of 13 to 25% [3, 20], and this might have been the case in autonomic settings as well.

In conclusion, while exerting a negative effect on the quality of clinical autonomic education in Europe, the COVID-19 pandemic promoted its digitalization and therefore fruition outside of autonomic referral centers. Likewise, the pandemic had a negative impact on clinical autonomic research and the scientific output of autonomic researchers. Nevertheless, it facilitated telemedicine implementation in clinical autonomic research, lowered the organizational barriers for networking among autonomic centers, and promoted digital literacy even among elderly individuals [24]. Altogether, digital communication tools provide novel educational and scientific opportunities, which the autonomic community should build upon beyond the COVID-19 pandemic horizon while taking into account legal and security aspects, as well as the needs of vulnerable patients with lower education and/or [23] cognitive disability [8].

### Supplementary Information

Below is the link to the electronic supplementary material.

Supplementary file1 (DOCX 108 kb)
