# Supplementary material for: EFAS/EAN survey on the influence of the COVID-19 pandemic on European clinical autonomic education and research
Source: Clin Auton Res. 2023 Oct 4;33(6):777–90. doi: 10.1007/s10286-023-00985-3 (PMC10751256; doi:10.1007/s10286-023-00985-3)
Supplement: Supplementary file 1 — Supplementary file1 (DOCX 108 kb) [file 10286_2023_985_MOESM1_ESM.docx]

**EFAS/EAN survey on the influence of the COVID-19 pandemic on European clinical autonomic education and research *Supplementary materials***

Table of contents

[EFAS/EAN web-based survey on the impact of the COVID-19 pandemic on European clinical autonomic education and research 2](#_Toc144743193)

[PubMed search terms used for assessing the pre-to pandemic publication performance in different Neurology subspecialties. 10](#_Toc144743194)

# EFAS/EAN web-based survey on the impact of the COVID-19 pandemic on European clinical autonomic education and research

1. **Identification and demographics of the survey participants**
2. Your name: ______________________________________
3. Department: _____________________________________________
4. Institution: _____________________________________________
5. City: ________________________________________________
6. Country: ________________________________________________
7. Mail contact: _____________________________________________
8. Would you like to be mentioned in the acknowledgements of the publication, which will summarize the results of the present survey?

Yes No

1. Your age (*dropdown question):*

*Options*: 20-29; 30-39; 40-49; 50-59; 60-69

1. Gender (*dropdown question)*:

*Options*: Female; Male; Other

1. Years into clinical practice (*dropdown question)*:

*Options*: Resident; junior consultant (0-4 years); consultant (5 – 9 years); senior consultant (10 – 19 years); > 20 years

1. **Characteristics of your ANS center** *(multiple choices are possible)*
   1. Equipment:

a.1. Cardiovascular

a.1.1. Testing for blood pressure

Invasive continuous (beat to beat)

Noninvasive continuous (beat to beat)

Volume clamp method

Pulse wave velocity

Other method: ________________

Riva-Rocci

a.1.2. Testing for heart rate

ECG

Other method: ________________

a.2. Sudomotor

Sympathetic skin response

Quantitative sudomotor axon reflex

Spontaneous Electrochemical Skin Conductance

Thermoregulatory Sweat test

Other method: ________________

a.3. Blood sampling

Catecholamine testing

Antibody testing

Other method: ________________

a.4. Histology

Skin biopsy for dermal fiber density

Other method: ________________

Other: __________

Other: ___________

Other: ___________

- 1. Staff (*No. per type*):

- Consultants: (*dropdown question* - *options*: 1 to 10)

- Residents: (*dropdown question* - *options*: 1 to 10)

- PhD: (*dropdown question* - *options*: 1 to 10)

- Visitors: (*dropdown question* - *options*: 1 to 10)

- Post-doc fellows: (*dropdown question* - *options*: 1 to 10)

- Medical students: (*dropdown question* - *options*: 1 to 10)

- Technicians: (*dropdown question* - *options*: 1 to 10)

- Nurses: (*dropdown question* - *options*: 1 to 10)

- Biomedical Engineer: (*dropdown question* - *options*: 1 to 10)

- Other: (*dropdown question* - *options*: 1 to 10)

- 1. Average No. of tilt test examination per year (pre-COVID): ___
  2. Could you describe the case mixture in your lab? (please add to 100%)

___ % reflex syncope

___ % neurogenic OH

___ % non-neurogenic OH

___ % initial OH

___ % delayed OH

___ % POTS

___ % cardiac syncope

___ % psychogenic pseudosyncope

___ % other causes of transient loss of consciousness

___ % negative

- 1. No. of inhabitants in the referral area (e.g. for the Innsbruck site: 750.000 inhabitants in Tirol) (*dropdown question):*

*Options*: ≤500.000; 500.000 – 1.000.000; 1.000.000 – 5.000.000; >5.000.000

- 1. How many tilt-test examinations did you perform during the pandemic? (March 1st 2020 - February 28th 2021): ____
  2. Do you have a dedicated ANS-outpatient clinic?

Yes No

- 1. Average No. of outpatient visits per year (pre-COVID): ____
  2. No. of outpatient visits during the pandemic (March 1st 2020 - February 28th 2021): ____
  3. Possibility of inpatient admission (yes/no)

Yes No

- 1. Average No. of inpatient admissions per year (pre-COVID): ____
  2. No. of inpatient admissions during the pandemic (March 1st 2020 - February 28th 2021): ____

1. **Are you involved in research activities?**

Yes No

1. Which is your research focus? (multiple choices are possible)

Reflex syncope

Cardiac syncope

Orthostatic hypotension

POTS

Psychogenic pseudosyncope

Other causes of transient loss of consciousness

Acute autonomic ganglionopathy

Sweating disorders

Urinary and bowel dysfunction

Movement disorders

Epilepsy

Multiple Sclerosis

Stroke

Headache

Sleep

Rare diseases

Which: __________

Others: ___________; ___________; ___________

**DEPARTMENT ORGANIZATION CHANGES**

1. **Was your autonomic function lab closed during the pandemic?**

Yes No

a. If yes, how long (cumulative No. of months from the begin of the pandemic to date)? (*dropdown question*)

*Options*: < 3; 3 – 6; 6 – 12; ≥ 12

1. **Was your autonomic outpatient clinic closed during the pandemic?**

Yes No

a. If yes, how long (cumulative No. of months from the begin of the pandemic to date)? (*dropdown question*)

*Options*: < 3; 3 – 6; 6 – 12; ≥ 12

1. **Was your autonomic inpatient clinic closed during the pandemic?**

Yes No

a. If yes, how long (cumulative No. of months from the begin of the pandemic to date)? (*dropdown question*)

*Options*: < 3; 3 – 6; 6 – 12; ≥ 12

**RESEARCH AND ACADEMIC ACTIVITIES**

1. **Education**
2. Are you involved in ANS educational activities?

Yes No

1. Did you have to stop in person classes during the COVID-19 pandemic?

Yes No

1. If yes, how long? (cumulative No. of months from the begin of the pandemic to date) (*dropdown question*)

*Options*: < 3; 3 – 6; 6 – 12; ≥ 12

1. Did you have to stop internships programs/practical training during the COVID- 19 pandemic?

Yes No

- 1. If yes, how long? (cumulative No. of months from the begin of the pandemic to date) (*dropdown question*)

*Options*: < 3; 3 – 6 (1 semester); 6 – 12 (2 semesters); ≥ 12 (more than 2 semesters)

1. Did your institution establish any distance learning models?

Yes No

- 1. If yes, which? *(multiple choices are possible)*

Online Lessons

Learning material to be downloaded

Webinars on demand

Others: ___________; ___________; __________

1. Are in person classes currently allowed in your institution?

Yes No

1. Are in-person internships programs/practical training currently allowed in your institution?

Yes No

1. How much did the COVID-19 pandemic negatively affect the quality of ANS education in your institution? (*Likert scale*)

No impact

Impact

Moderate impact

Major impact

1. Any suggestion how to fill the gap in education? (open question)
2. **Research**

a. Did you have to stop patients’ recruitment into clinical trials during the COVID- 19 pandemic?

Yes No

b. If yes, how long (cumulative No. of months from the begin of the pandemic to date)? (*dropdown question*)

*Options*: < 3; 3 – 6; 6 – 12; ≥ 12

c. Did you amend research protocols to integrate telemedicine study visits?

Yes No

1. Did you lose financial support due to the COVID pandemic?

Yes No

1. Did you experience any delay in your project timelines?

Yes No

- 1. If yes, did you run out of budget because of these delays?

Yes No

1. Did you experience difficulties in running multi-center projects?

Yes No

1. Did you have any international exchange program, which was stopped due to the pandemic?

Yes No

1. Did you have more or less time for grant writing:
2. During the 1st wave of the pandemic (spring 2020)?

More Less

1. During the following waves of the pandemic?

More Less

1. Did you have more or less time for paper writing:
2. During the 1^st^ wave of the pandemic (spring 2020)?

More Less

1. During the following waves of the pandemic?

More Less

1. **Lessons we’ve learnt from the pandemic**

Which of the changes determined by the COVID-19 pandemic should be kept in the future for:

Autonomic education? (open question)

A: _____________________________________________________________

Autonomic research? (open question)

A: _____________________________________________________________

# PubMed search-terms used for assessing the pre-to pandemic publication performance in different neurology subspecialties.

- *Autonomic nervous system*: autonomic nervous system OR orthostatic hypotension OR syncope OR postural orthostatic tachycardia syndrome OR sudomotor dysfunction;
- *Dementia*: dementia OR Alzheimer's disease OR frontotemporal dementia OR dementia with Lewy bodies;
- *Epilepsy*: epilepsy OR seizure OR antiepileptic drugs OR epilepsy surgery;
- *Demyelinating disorders:* multiple sclerosis OR demyelination OR neuromyelitis optica spectrum disorder;
- *Neuromuscular Disorders:* polyneuropathy OR Guillain Barre syndrome OR Myasthenia gravis OR myopathy;
- *Movement disorders*: movement disorders OR Parkinson's disease OR Friedreich's ataxia OR Huntington's disease;
- *Stroke*: stroke OR subarachnoid hemorrhage OR carotid arteries ultrasound OR transcranial doppler ultrasound;
- *Headache*: migraine OR headache OR trigeminal neuralgia;
- *Neurocritical Care*: neuromonitoring OR intracranial hemorrhage OR traumatic brain injury OR concussion;
- *Clinical Neurophysiology*: evoked potentials OR electroencephalography OR electroneurography OR polysomnography.
